# Functional Language Shift to the Right Hemisphere in Patients with Language-Eloquent Brain Tumors

**DOI:** 10.1371/journal.pone.0075403

**Published:** 2013-09-17

**Authors:** Sandro M. Krieg, Nico Sollmann, Theresa Hauck, Sebastian Ille, Annette Foerschler, Bernhard Meyer, Florian Ringel

**Affiliations:** 1 Department of Neurosurgery; Klinikum rechts der Isar, Technische Universität München, Germany; 2 Section of Neuroradiology; Klinikum rechts der Isar, Technische Universität München, Germany; University of Ulm, Germany

## Abstract

**Objectives:**

Language function is mainly located within the left hemisphere of the brain, especially in right-handed subjects. However, functional MRI (fMRI) has demonstrated changes of language organization in patients with left-sided perisylvian lesions to the right hemisphere. Because intracerebral lesions can impair fMRI, this study was designed to investigate human language plasticity with a virtual lesion model using repetitive navigated transcranial magnetic stimulation (rTMS).

**Experimental design:**

Fifteen patients with lesions of left-sided language-eloquent brain areas and 50 healthy and purely right-handed participants underwent bilateral rTMS language mapping via an object-naming task. All patients were proven to have left-sided language function during awake surgery. The rTMS-induced language errors were categorized into 6 different error types. The error ratio (induced errors/number of stimulations) was determined for each brain region on both hemispheres. A hemispheric dominance ratio was then defined for each region as the quotient of the error ratio (left/right) of the corresponding area of both hemispheres (ratio >1  =  left dominant; ratio <1  =  right dominant).

**Results:**

Patients with language-eloquent lesions showed a statistically significantly lower ratio than healthy participants concerning “all errors” and “all errors without hesitations”, which indicates a higher participation of the right hemisphere in language function. Yet, there was no cortical region with pronounced difference in language dominance compared to the whole hemisphere.

**Conclusions:**

This is the first study that shows by means of an anatomically accurate virtual lesion model that a shift of language function to the non-dominant hemisphere can occur.

## Introduction

The cortical distribution and variability of human language representation has been widely examined. Current knowledge is mainly based on functional MRI (fMRI) studies [1], [2], [3] and on intraoperative language mapping by bipolar direct cortical stimulation (DCS) during awake surgery for the left hemisphere [4], [5], [6]and, but also for the right hemisphere [7]. Although intraoperative mapping is highly reliable, it does not allow for the examination of language distribution in the healthy brain. Navigated transcranial magnetic stimulation (nTMS) is increasingly used for preoperative mapping of the primary motor cortex, and a good correlation of preoperative nTMS and intraoperative DCS motor maps has been repeatedly reported [8], [9], [10]. This method allows the transcranial non-invasive magnetic induction of an electric field within the cortex. By single pulse stimulation, it can elicit muscular evoked potentials within the motor cortex. But by applying pulse trains, we can also depolarize neurons and therefore cause a “virtual lesion” for the 1–4 seconds of stimulation [11], [12]. By combining it with an object-naming task, this repetitive TMS has been repeatedly used for disturbing language function and determination of language lateralization in the past [11], [12], [13]. Moreover, by combining repetitive TMS with a navigation system, we can even specifically define cortical regions, which are vulnerable to repetitive nTMS (rTMS) and therefore considered language-eloquent. It has thus been shown that rTMS during an object-naming task allows us to map the cortex for language eloquent regions [14].

The right hemisphere was shown to participate in language function not only in healthy participants [15], [16], [17], [18], but also in patients after left-hemispheric stroke [19], [20] or brain tumors [21], [22], [23], [24], [25], [26]. These reports used a variety of methods, including neuropsychological assessment, non-navigated TMS, and mainly fMRI, which is frequently impaired by intracerebral tumors and ischemic lesions. For brain-tumor patients, the right inferior frontal gyrus (IFG) was also shown to be involved in language production in a previous study using non-navigated TMS combined with an object-naming task [25], [26]. However, in these previous works, the extent of language lateralization was not investigated with a high spatial resolution concerning the exact location of the gyri involved in changed lateralization of language function. In a recently performed study, our group showed a high sensitivity of preoperative language mapping by rTMS compared to intraoperative DCS during awake surgery when the human cortex was divided by the sections according to the cortical parcellation system [5], [27], [28]. Moreover, we showed the superiority of rTMS language mapping compared to fMRI in a glioma patient in terms of language lateralization [29].

Because a change in language lateralization would increase surgical options for patients with left-sided perisylvian tumors, it would represent a new approach in surgical neurooncology. Thus, this study was designed to find evidence for the extent of change in language lateralization for every single gyri via a virtual lesion model using rTMS of both hemispheres.

## Materials and Methods

### Ethics approval

The experimental protocol was approved by the ethics committee of the Technical University Munich (registration number: 2793/10) in accordance with the declaration of Helsinki. All volunteers and patients provided written informed consent prior to MR imaging.

### Study design

The study was designed as prospective and non-randomized.

### Study participants

Between April 2011 and October 2012, 15 patients (14 right-handed, 1 left-handed; 8 male, 7 female) and 50 healthy volunteers (all right-handed; 25 male, 25 female) underwent rTMS language mapping of both hemispheres. By using such a large and homogenous control group, we are able to provide exact data on healthy subjects for further comparison with patient data. Table 1 shows the properties of the two groups of participants. In the 15 patients, there were 2 cavernomas (anG, opercular), 1 temporal astrocytoma WHO°II, 2 astrocytoma WHO°III of the anG, 1 temporal astrocytoma WHO°III, 4 opercular GBM, 4 temporal GBM, and 1 GBM of the anG. Table 2 gives a detailed description of main tumor location and further affected gyri or fascicles by displacement or infiltration. German was the primary language of all participants. The inclusion criteria for all participants were:

**Table 1:** Mapping parameters.

group		healthy subjects	patients	p
Age	(mean ± SD)	25.9±5.4	43.9±10.6	p<0.0001
Pain (VAS) (Mean ± SD)	convexity	2.0±1.3	1.9±1.7	n.s.
Pain (VAS) (Mean ± SD)	temporal	5.3±1.7	4.2±2.1	n.s.
Representative correct baseline pictures	(out of 131)	111.2±5.2	87.3±22.2	p<0.05
RMT	(% Output) (mean ± SD)	36.2±6.6	35.6±8.4	n.s.
mapping intensity	(% MT) (mean ± SD)	101.4±5.1	102.9±10.8	n.s.
	5 Hz, 5 pulses	17 (34.0%)	10 (66.7%)	
most comfortable	7 Hz, 5 pulses	18 (36.0%)	2 (13.3%)	n.s.
	7 Hz, 7 pulses	15 (30.0%)	3 (20.0%)	

Stimulation parameters used in the study including group and pain score, according to the visual analogue scale (VAS). RMT  =  resting motor threshold (stimulator output); Hz  =  stimulation train frequency; # pulses  =  number of pulses in train; int %  =  stimulation intensity (of maximum stimulator output). The designation n.s.  =  statistically not significant (p>0.05).

**Table 2:** Tumor location.

Patients	Tumor type	Age (years)	RMT (%)	Main tumor location	Infiltrated structures	Displaced structures
M3	AA	53	38	pMTG	**pSTG, anG**	**FT**
M4	GBM	43	58	opIFG	**vPrG, pMFG, FT**	**aSTG**
M5	GBM	51	25	anG	**pSMG, pSTG, FT**	**-**
M6	GBM	40	39	pSTG	**pSMG, mSTG, pMTG, FT**	**aSMG**
M7	C	34	43	mMFG	**-**	**-**
M9	GBM	33	37	mSTG	**aSTG, pSTG, mMTG, FT**	**vPoG, opIFG**
M10	GBM	53	41	opIFG	**vPrG, pMFG, FT**	**aSTG**
M11	GBM	43	21	opIFG	**vPrG, mPrG, pMFG, FT**	**aSTG**
F1	AA	29	34	anG	**pSMG, pSTG, pMTG**	**-**
F5	DA	63	36	pSTG	**mSTG, pMTG**	**-**
F6	GBM	47	30	pMTG	**pSTG, anG**	**FT**
F7	GBM	56	31	pMTG	**pSTG, anG**	**FT**
F8	C	32	33	anG	**-**	**-**
F10	GBM	52	33	opIFG	**pMFG, mMFG, trIFG, vPrG**	**aSTG, polSTG**
F11	AA	30	35	anG	**pSMG, pSTG, pMTG**	**FT**

Detailed description of main tumor location and further affected gyri or fibre tracts (FT) by displacement or infiltration for male (M) and female (F) patients. Resting motor threshold (RMT) is also provided. Abbreviations: AA  =  anaplastic astrocytoma WHO grade III, GBM  =  glioblastoma WHO grade IV, C  =  cavernoma, DA  =  diffuse astrocytoma WHO grade II.

1. age >18 years; and
2. written informed consent.

The exclusion criteria for all participants were general TMS exclusion criteria, such as pacemaker or cochlear implant [30]. Additional exclusion criteria for healthy volunteers were:

1. previous seizures;
2. bilateral handedness;
3. second mother tongue;
4. pathological findings on cranial MRI;
5. aberrant medical history;
6. developmental language deficits; and
7. neurological impairments.

### Navigational MRI scan

For neuronavigation, the rTMS system requires a 3D MRI dataset for anatomical co-registration. Subsequent to informed consent, all participants underwent a navigational MRI scan on a 3 Tesla MR scanner (Achieva 3T, Philips Medical Systems, The Netherlands B.V.) using an 8-channel phased array head coil. Our protocol consisted of a three-dimensional (3D) gradient echo sequence (TR/TE 9/4 ms, 1 mm^3^ isovoxel covering the whole head, 6 minutes 58 seconds acquisition time) without (for volunteers) or with (for patients) intravenous contrast administration for anatomical co-registration. The 3D dataset was then transmitted to the nTMS system using DICOM standard.

### Language mapping by rTMS

#### Language mapping setup

The following experimental setup was applied to all participants without differences between the groups. Language mapping was performed with the Nexstim eXimia NBS system 4.3 with the NexSpeech® module (Nexstim Oy, Helsinki, Finland) as documented earlier [27], [29]. Briefly, the 3D T1-weighted MRI of each participant was used as an anatomical reference and registered to the participant's brain to visualize the exact brain area receiving rTMS pulses by a stereotactic camera to track coil position [10]. As reported earlier, the stimulating coil induces an electric field within the brain, which is represented by the software as a 3D reconstruction [31], [32], [33]. The intracranial stimulation points are then saved for later analysis [33]. Immediately before language mapping, the Resting Motor Threshold (RMT) was defined by motor mapping of the cortical representation of the contralateral hand area at the left hemisphere (right abductor pollicis brevis muscle) [8]. The RMT of each participant is a measure for motor cortex excitability and therefore was used as a basic value for the following rTMS examination [14], [27], [29]. Because object-naming tasks are also used for intraoperative language mapping, they were used in this study to identify language-eloquent cortical regions by causing a virtual functional lesion by rTMS as described and analyzed earlier [14], . One hundred thirty-one colored pictures of common objects were displayed at an inter-picture interval (IPI) of 2.5 s. Frequency and intensity of the rTMS were personalized based on a previously published protocol [27], [29]:

1. RMT on the left hemisphere was determined thoroughly;
2. a train of 5-7 rTMS bursts was administered to vPrG and opIFG:
  a) 5 Hz, 5 pulses, 100% RMT;
  b) 7 Hz, 5 pulses, 100% RMT;
  c) 7 Hz, 7 pulses, 100% RMT;
3. the setup (a-c) that caused the highest error rate (number of errors/number of stimulations) was identified by the volunteer's and examiner's impression and in unclear cases supported by video analysis;
4. if there was no clear difference in the effect on language, the most comfortable frequency was chosen;
5. if naming was not interrupted clearly by rTMS, the intensity was increased to 110–120% RMT and step 1 was repeated; and
6. if significant pain was reported, the stimulation intensity was decreased to 80–90% RMT to avoid any discomfort that might interfere with the consecutive response evaluation [13]. This was also done if 100% RMT was painful.

Moreover, after step 2, every participant was asked to report the most comfortable stimulus sequence and the rate of discomfort or pain according to the visual analogue scale (VAS) from 0 (no pain) to 10 (maximum pain).

Minimum electric field strength was not lower than 55 V/m at the cortical region of interest and ranged between 55–80 V/m in all participants during the entire mapping. During the object-naming task, the pictures had to be named immediately upon presentation. Presentation onset was 300 ms prior to rTMS pulses based on our present knowledge of naming-related cortical activity reported in magnetoencephalography (MEG) and TMS studies [37], [38], [39]. To assure objective and detailed analysis, the object-naming baseline performance and the mapping were digitally video-recorded [14], [27].

#### rTMS mapping procedure

The procedure was performed as reported earlier and included two consecutive baseline tests to document individual differences in the participant’s vocabulary [14], [27], [29]. During baseline and mapping, the images were randomly displayed on a screen in front of the participant, who named them in German as quickly and precisely as possible. Starting at the mSFG as the most comfortable site with the lowest pain intensity, the stimulation coil was then randomly moved after each image in 10 mm steps over both hemispheres and placed tangential to the skull in strict anterior-posterior field orientation to achieve maximum field induction [12], [13], [14]. All sites were stimulated 3 times each and were not targeted consecutively. Language mapping required 60–90 minutes per participant.

Still, we had to restrict the spatial extent of stimulation due to unacceptable pain, especially due to direct stimulation of oculomotor muscles when applying rTMS to orIFG, polSTG, polMTG, aMTG, polSFG, polMFG, and polIFG (Table 3). Due to the increasing distance between skin and brain, stimulation intensity decreased below 50 V/m at the ITG. Therefore, ITG was not mapped.

**Table 3:** Cortical parcellation system.

Abbreviation	Anatomy
aITG	Anterior inferior temporal gyrus
aMFG	Anterior middle frontal gyrus
aMTG	Anterior middle temporal gyrus
anG	Angular gyrus
aSFG	Anterior superior frontal gyrus
aSMG	Anterior supramarginal gyrus
aSTG	Anterior superior temporal gyrus
dLOG	Dorsal lateral occipital gyrus
dPoG	Dorsal post-central gyrus
dPrG	Dorsal pre-central gyrus
mITG	Middle inferior temporal gyrus
mMFG	Middle middle frontal gyrus
mMTG	Middle middle temporal gyrus
mPoG	Middle post-central gyrus
mPrG	Middle pre-central gyrus
mSFG	Middle superior frontal gyrus
mSTG	Middle superior temporal gyrus
opIFG	Opercular inferior frontal gyrus
orIFG	Orbital part of the inferior frontal gyrus
pITG	Posterior inferior temporal gyrus
pMFG	Posterior middle frontal gyrus
pMTG	Posterior middle temporal gyrus
polIFG	Polar inferior frontal gyrus
polITG	Polar inferior temporal gyrus
polLOG	Polar lateral occipital gyrus
polMFG	Polar middle frontal gyrus
polMTG	Polar middle temporal gyrus
polSFG	Polar superior frontal gyrus
polSTG	Polar superior temporal gyrus
pSFG	Posterior superior frontal gyrus
pSMG	Posterior supramarginal gyrus
pSTG	Posterior superior temporal gyrus
SPL	Superior parietal lobe
trIFG	Triangular inferior frontal gyrus
vLOG	Ventral lateral occipital gyrus
vPoG	Ventral post-central gyrus
vPrG	Ventral pre-central gyrus

Anatomical names and abbreviations are according to Corina et al. 2005.

### Data analysis

The recorded mapping data were examined post-hoc and blinded to the participant, group, and tumor location as done previously [14], [27]. The baseline performance was analyzed first. Then, any disturbance of language processing at the object-naming task during stimulation was compared with the corresponding baseline response. Moreover, the cortical stimulation sites were hidden during video analysis. All observed errors were then categorized, as has been extensively outlined in earlier publications [5], [27], [29]: no-response errors, performance errors, hesitations, neologisms, semantic paraphasias, phonologic paraphasias, and circumlocution errors. Because hesitation errors represent a very inaccurate definition of an error category due to the lack of standardized latency recordings, we also defined an additional category: all errors without hesitations. Moreover, the category “all errors” was also defined as a sum of all categories.

### Cortical map of evoked errors

#### Anatomical localization

**Figure 1:**
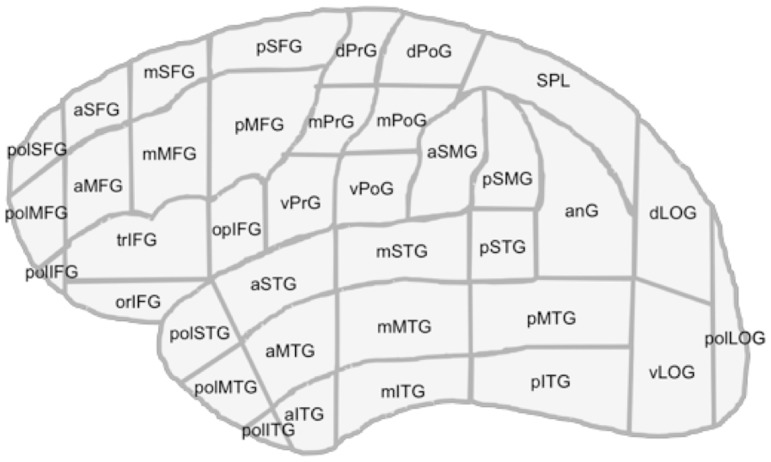
Cortical parcellation system. Anatomical areas, as described in Corina et al. 2005.

For anatomy-related data analysis, we used the cortical parcellation system (CPS), which was also used in previous studies on language distribution during awake surgery [5], [28]. The cortex is parcellated into 37 individual anatomical regions, and the cortical gyri belonging to these anatomical CPS subregions were identified from 3D MRIs (Table 3; Figure 1). This approach allows statistical analysis of error frequency and comparison of the data between individual participants and over the entire studied cohort.

#### Stimulation assessment

To analyze whether rTMS elicited language deficits in an individual brain region, the following definitions for region positivity and negativity were used: (1) positive brain region: A region was considered to give rise to language deficits if any of the trains delivered to the region elicited naming errors, regardless of the error type; and (2) negative brain region: A brain region was considered not to give rise to language deficits if the region had been stimulated with at least one stimulation train and no language deficits of any error type were generated.

### Hemispheric dominance ratio

An error ratio was defined as the number of induced errors per number of applied rTMS trains for each error category and each CPS region of both hemispheres. Moreover, a hemispheric dominance ratio (HDR) was defined as the quotient of the left-sided divided by the right-sided error ratio for the corresponding left and right CPS region. A HDR >1 means left-sided dominance; HDR <1 means right-sided language dominance.

### Statistical analysis

For testing the distribution of attributes between the groups, a Chi-square test was performed. Differences between 2 groups were tested by independent samples t-test. All results are presented as mean ± standard deviation (SD). Ranges are also reported in the text (GraphPad Prism 5.0c, La Jolla, CA, USA); p<0.05 was considered significant.

## Results

### Stimulation-related discomfort

The stimulation was generally well tolerated by all participants. The mean VAS score for maximum painful stimuli was highly comparable in the two groups and did not differ between the hemispheres (Table 1). No participant requested reduction of the stimulation intensity due to discomfort or pain in any group. Moreover, no adverse events were observed.

### rTMS mapping parameters and errors

Each rTMS train consisted of 5–7 pulses given at rates between 5–7 Hz (Table 1). There were no differences in the best suitable stimulation setup between genders (Table 1). The number of stimulated sites per hemisphere varied due to head size, tumor location, tumor size, and the participant’s cooperation. Still, there is no significant difference in the number of stimulated sites between groups or hemispheres. In healthy volunteers, the left hemisphere was stimulated at 396.1±111.9 sites per participant (range: 258–789 sites). During stimulation, 82.5±44.4 naming errors ( = all errors) (range: 12–241 errors), 21.8±21.5 no response errors (range: 0–107 errors), 25.2±22.5 performance errors (range: 1–124 errors), 30.7±16.4 hesitations (range: 3–84 hesitations), and 2.9±7.4 neologisms (range: 0–48 neologisms) were observed. In patients, the left hemisphere was stimulated at 442.8±167.9 sites per patient (range: 222–675 sites). During stimulation, 96.3±55.0 naming errors ( = all errors) (range: 18–180 errors), 38.9±40.4 no response errors (range: 0–155 errors), 19.6±22.2 performance errors (range: 0–68 errors), 33.1±22.6 hesitations (range: 2–76 hesitations), and 2.3±2.6 neologisms (range: 0–7 neologisms) were observed.

In healthy volunteers, the right hemisphere was stimulated at 196.8±54.4 sites (range: 81–402 sites). During stimulation, 29.7±17.5 naming errors ( = all errors) (range: 2–87 errors), 9.6±9.3 no response errors (range: 0–37 errors), 7.9±8.1 performance errors (range: 0–40 errors), 11.0±6.8 hesitations (range: 0–31 hesitations), and 0.6±1.1 neologisms (range: 0–6 neologisms) were observed. In patients, the right hemisphere was stimulated at 151.8±40.1 sites (range: 99–231 sites). During stimulation, 31.7±17.7 naming errors ( = all errors) (range: 5–69 errors), 14.8±15.5 no response errors (range: 0–54 errors), 6.0±6.7 performance errors (range: 0–21 errors), 8.9±7.4 hesitations (range: 2–27 hesitations), and 0.7±1.1 neologisms (range: 0–4 neologisms) were observed.

### Distribution of the hemispheric dominance ratio

#### Whole hemispheres

**Figure 2:**
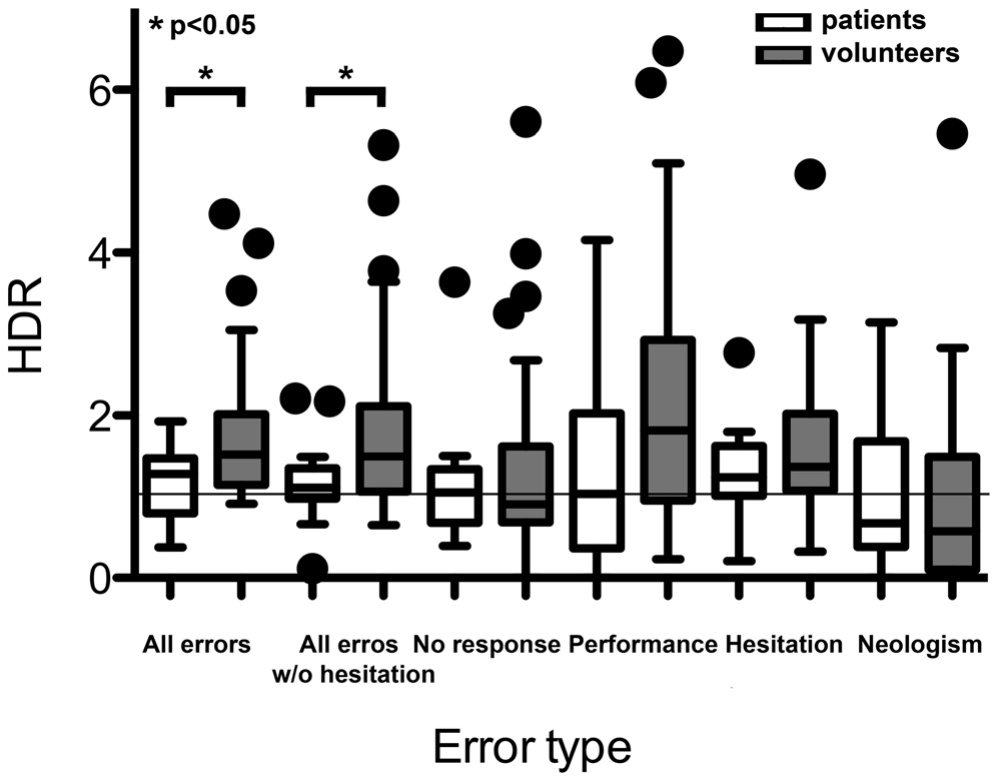
Hemispheric dominance ratio for different errors. The graph shows the hemispheric dominance ratio (HDR; quotient of the error rate for the left and right hemisphere) as a box plot for the different error types. A hemispheric dominance ratio >1 means left-sided dominance, and <1 means right-sided language dominance.

Overall, patients with language-eloquent tumors showed a significantly lower HDR than healthy participants concerning “all errors” (patients: 1.24±0.46; healthy participants: 1.76±0.80; p<0.05) and “all errors without hesitations” (patients: 1.18±0.54; healthy participants: 1.79±1.03; p<0.05), which indicates a higher participation of the right hemisphere in language function (Table 4; Fig. 2). All other single error categories failed to show statistical differences between the groups.

**Table 4:** Hemispheric dominance ratio.

	healthy subjects		patients		p
	mean	SD	mean	SD	
**All errors**	1.76	0.80	1.24	0.46	0.0349
**All errors without hesitations**	1.79	1.03	1.18	0.54	0.0482
**No response**	1.46	1.48	1.17	0.82	n.s.
**Performance**	2.14	1.45	1.33	1.18	n.s.
**Hesitation**	1.78	1.52	1.93	2.64	n.s.
**Neologism**	1.09	1.40	1.03	1.10	n.s.

Summary of the hemispheric dominance ratio as a quotient of the error rate (fraction of the absolute number of errors and number of stimulations per region) for the corresponding left and right CPS regions separated in the different error types. A hemispheric dominance ratio >1 means left-sided dominance, and <1 means right-sided language dominance. n.s.  =  statistically not significant (p>0.05).

#### CPS subregions

**Figure 3:**
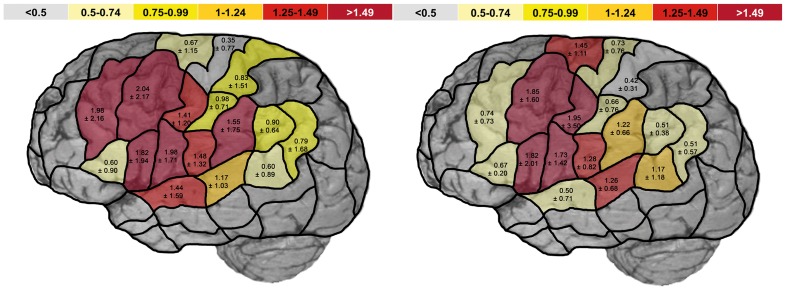
Hemispheric dominance ratio of all naming errors. The scheme shows the mean hemispheric dominance ratio (HDR) ± standard deviation of all naming errors in healthy volunteers (left) and patients (right) for each CPS region. The hemispheric dominance ratio is the quotient of the error rate (fraction of the absolute number of errors and number of stimulations per region) for the corresponding left and right CPS regions. A hemispheric dominance ratio >1 means left-sided dominance, and <1 means right-sided language dominance.

**Figure 4:**
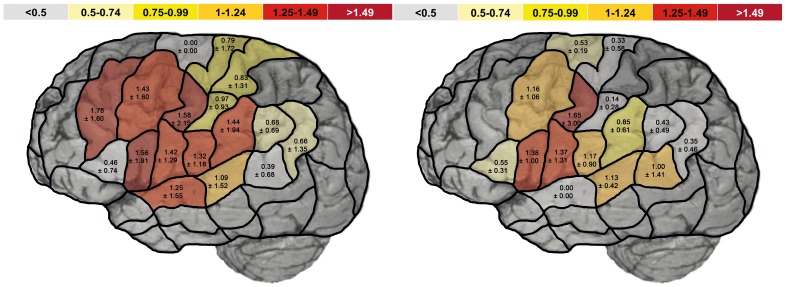
Hemispheric dominance ratio of all naming errors without hesitations. The scheme shows the mean hemispheric dominance ratio (HDR) ± standard deviation of all naming errors without hesitations in healthy volunteers (left) and patients (right) for each CPS region. The hemispheric dominance ratio is the quotient of the error rate (fraction of the absolute number of errors and number of stimulations per region) for the corresponding left and right CPS regions. A hemispheric dominance ratio >1 means left-sided dominance, and <1 means right-sided language dominance.

When taking a closer look at each subregion, we could not show any statistically significant difference in HDR between the two groups for the separate corresponding CPS regions of both hemispheres and the different error categories (Figures 3 and 4). Yet, figures 3 and 4 still show visual differences between both groups even when not reaching statistical significance, which might has to be attributed to the small patient group. However, when analyzing and visualizing the HDR for the category “all errors”, we observed a decreased HDR in patients compared to healthy participants in favor of right-sided language dominance at mMFG (patients: 0.74±0.73; healthy participants: 1.98±2.16), aSTG (patients: 0.50±0.71; healthy participants: 1.44±1.59), aSMG (patients: 1.22±0.66; healthy participants: 1.55±1.75), pSMG (patients: 0.51±0.38; healthy participants: 0.90±0.64), and anG (patients: 0.51±0.57; healthy participants: 0.79±1.68) (Figure 3). Yet despite showing a trend (p<0.15), these regions also failed to show a statistically significant difference.

The HDR for the category “all errors without hesitation” was decreased in patients compared to healthy participants in favor of right-sided language dominance at mMFG (patients: 0.22±0.0; healthy participants: 1.78±1.60; not significant), pMFG (patients: 1.16±1.06; healthy participants: 1.43±1.60; not significant), pSTG (patients: 1.13±0.42; healthy participants: 1.09±1.52; not significant), aSMG (patients: 0.85±0.61; healthy participants: 1.43±1.94; not significant), mPoG (patients: 0.14±0.28; healthy participants: 0.97±0.93; p<0.05), and anG (patients: 0.35±0.46; healthy participants: 0.66±1.35; not significant) (Figure 4).

Nevertheless, with the exception of mPoG, no CPS region showed a pronounced difference in language dominance compared to the whole hemisphere. Thus, language switch seems to show a more diffuse rather than focused pattern. Moreover, we were not able to reveal any correlation between laterality and aphasia severity, lesion size, lesion site, or the subtype of lesion.

## Discussion

All patients were proved by awake surgery to have left-sided language function. Concerning rTMS-induced language impairment, a HDR was defined for each CPS region. Overall, patients with language-eloquent tumors showed a statistically significantly lower HDR than healthy participants concerning “all errors” and “all errors without hesitations”, which is a sign of a higher participation of the right hemisphere in language function.

Recent models of language production during an object-naming task suggest a multi-stage word production process. In short, phonological representations are accessed and information is transmitted to working memory. The representations are then converted into a series of phonological targets at the prearticulatory stage, and after that, a motor command is initiated [39], [40], [41]. Moreover, tasks like picture naming and word generation may involve rather different concepts and consequently only enter a common pathway from the point of concept-based lexical retrieval onward [39]. There are several studies that used lesion-based approaches to identify truly essential cortical regions for word production, instead of regions, which are only involved but not essential. In these works, transient lesions consistently interfered with object naming and therefore demonstrated that the core areas for language processing during an object-naming task can be identified [4], [6], [42]. Such regions were left-sided opIFG, precentral gyrus, mSTG, pSTG, and middle temporal gyri [39]. Nonetheless, these cerebral regions do not include all cortical areas, which participate in conceptual processing [43], [44]. However, when comparing such lesion-based investigations with hemodynamic studies such as fMRI, these approaches did not routinely reveal cortical language areas in the inferior parietal cortex and adjacent to intracerebral lesions, such as tumors or vascular malformations, which are known to impair tissue oxygenation and therefore blood oxygenation level dependence (BOLD) [20], [29], [45], [46], [47]. In the patient group, one patient was left-handed. However, all enrolled patients were proven to have left-sided language function as revealed by awake surgery and DCS mapping. In the volunteer group, however, all participants were purely right-handed and were also mapped in their mother tongue. Thus, we can state that all enrolled participants were left dominant in terms of language function. However, the patient group was significantly older (p<0.0001) than the healthy participants, which has to be considered a limitation of our study. The literature has documented that aging can affect hemispheric laterality in general, and language production in particular [48], [49], [50]. Yet, the patients in these cited studies were significantly older than those in our patient cohort, and none of our patients showed ischemic lesions within the white matter, which might be one reason for the observed changes with age in the above-cited studies. Moreover, the participants in the two groups were old enough that cortical development was finished. And since all enrolled patients did not suffer from any additional supratentorial pathology such as ischemia, it is unlikely that the age difference would impair the results of this study. Additionally, although RMT was shown to change with age, our groups did not differ in RMT, which can be judged another argument for the comparability of the two groups (Table 1) [51].

Likewise, the patient group showed a lower number of correctly named baseline pictures as a sign for aphasia and therefore left-sided language production in these patients (Table 1). But because the HDR is a quotient that takes the error ratio of both hemispheres into consideration, we are able to encounter any aphasia in the patient group.

As another limitation of the presented study, the patient group is small due to the rare occurrence of such patients but might be a reason that we only partially observed significant differences between both groups. However, we were still able to actually show significant differences between both groups even with this small sample size.

The observed shift of language function showed a more diffuse rather than focused pattern, although we were able to show a trend to a decreased HDR in some areas of the CPS, although without pronunciation of anterior or posterior cortical language areas (Figures 3 and 4). Moreover, we have to mention that the precentral gyrus showed a comparable HDR in the two groups in favor of the left hemisphere. Concerning the precentral gyrus, we are aware that this cortical area is eloquent for speech rather than language processing. This differentiation is useful for separating the motor part of language from conceptual, phonological, and semantic processing of language [39]. This points out that our rTMS protocol is actually able to identify cortical regions that are essential in language and not only in speech processing.

Furthermore, we were able to show that a virtual lesion to all corresponding right cortical regions caused all kinds of errors, especially no response and performance errors (Table 4). As also mentioned in previous works on DCS mapping in brain tumor patients, these evoked errors (no response and performance errors) may represent interference of processing information between phonological representations and articulatory motor representations of speech rather than actual language function, as also reported for the left hemisphere [5].

In our study, language reorganization was observed as a partial shift of language function to the right hemisphere, as also shown in other studies [21], [52], [53], [54]. However, these studies used imaging techniques such as MEG and fMRI rather than the lesion-based approaches used in our study [15], [18]. Our data were consistent with previous results of other studies, suggesting an interhemispheric reorganization (Figure 3) [24], [55], [56]. However, some studies did not observe any modification of language lateralization, especially in epilepsy patients. In these studies, temporal lobe activity differed between the different task types, showing that activation of these regions might be changed by linguistic processing due to epileptic activity but without inducing contralateral language function [57], [58]. It was also reported that patients with left-sided temporal lobe epilepsy suffered from a purely phonological deficit prior to lesionectomy, and it was shown that this deficiency was associated with right-hemispheric temporal activity in fMRI. This observation provides evidence that the shift to the functional right hemisphere compensated for the phonological deficit of the corresponding left-sided region [22].

Additionally, our data strictly contradict the broadly represented concept of limited plasticity in adults, which is also refuted by other studies [59], [60], [61]. However, we have to emphasize that language reorganization might not always be able to compensate all functional impairment per se and may therefore not lead to an absence of language deficits because our patients also showed aphasia to some degree in many cases (Table 3). On the contrary, there are reports that a partial shift of language function to the right hemisphere might even reduce language abilities in these patients [62]. However, there are even studies on complete resection of the left-sided Wernicke’s area without resulting aphasia [63]. Such astonishing results might be based not only on ipsilateral, but also on contralateral shift of language function within the human brain. When reviewing the literature, we also have to take into account that functional reorganization in terms of language lateralization varies significantly dependent on the executed language test [15].

Nevertheless, when we discuss language function, we have to mention another limitation of this study. By using only an object-naming task, we are only able to study the production of words but not the production of sentences, comprehension, or repetition.

To summarize, our study provides highly interesting data because it is the first lesion-based study that actually proves language plasticity as a shift to the non-dominant hemisphere by an anatomically traceable method [25], [26]. However, the clinical application of these findings will be revealed in the future. On the one hand, it is gratifying that lesion-based and BOLD-based imaging modalities lead to comparable findings, which many neuroscientists still doubt. On the other hand, the degree of language shift in brain tumor patients could be measured by rTMS in the future to indicate the potential of a more extensive tumor resection within perisylvian language regions, which has already been shown to be feasible in low-grade gliomas by neurosurgical authors [64], [65], [66].

Additionally, we showed that language mapping via rTMS and an object-naming task reveals that a widespread distribution of cortical regions is involved in the network of human language processing—even in the contralateral hemisphere.

## Conclusions

Despite its limitations, especially in terms of age difference of both groups, this study significantly contributes to the evidence that lesions within language-eloquent brain can induce plasticity as a shift of language function to the non-dominant hemisphere although this plasticity seems to show a more diffuse pattern.

## References

[1] Griffiths JD, Marslen-Wilson WD, Stamatakis EA, Tyler LK (2012) Functional Organization of the Neural Language System: Dorsal and Ventral Pathways Are Critical for Syntax. Cereb Cortex. 10.1093/cercor/bhr386 PMC3601415 22275482

[2] McGraw P, Mathews VP, Wang Y, Phillips MD (2001) Approach to functional magnetic resonance imaging of language based on models of language organization. Neuroimaging Clin N Am 11: : 343–353, x. 11489743

[3] Hund-Georgiadis M, Lex U, von Cramon DY (2001) Language dominance assessment by means of fMRI: contributions from task design, performance, and stimulus modality. J Magn Reson Imaging 13: 668–675. 11329187 10.1002/jmri.1094

[4] Ojemann G, Ojemann J, Lettich E, Berger M (1989) Cortical language localization in left, dominant hemisphere. An electrical stimulation mapping investigation in 117 patients. J Neurosurg 71: 316–326. 2769383 10.3171/jns.1989.71.3.0316

[5] Corina DP, Loudermilk BC, Detwiler L, Martin RF, Brinkley JF, et al (2010) Analysis of naming errors during cortical stimulation mapping: implications for models of language representation. Brain Lang 115: 101–112. 20452661 10.1016/j.bandl.2010.04.001 PMC3247200

[6] Sanai N, Mirzadeh Z, Berger MS (2008) Functional outcome after language mapping for glioma resection. NEnglJMed 358: 18–27. 10.1056/NEJMoa067819 18172171

[7] Chang EF, Wang DD, Perry DW, Barbaro NM, Berger MS (2011) Homotopic organization of essential language sites in right and bilateral cerebral hemispheric dominance. J Neurosurg 114: 893–902. 21235314 10.3171/2010.11.JNS10888

[8] Picht T, Mularski S, Kuehn B, Vajkoczy P, Kombos T, et al (2009) Navigated transcranial magnetic stimulation for preoperative functional diagnostics in brain tumor surgery. Neurosurgery 65: 93–98. 19935007 10.1227/01.NEU.0000348009.22750.59

[9] Krieg SM, Shiban E, Buchmann N, Meyer B, Ringel F (2012) Presurgical navigated transcranial magnetic brain stimulation for recurrent gliomas in motor eloquent areas. Clin Neurophysiol. 10.1016/j.clinph.2012.08.011 22986282

[10] Krieg SM, Shiban E, Buchmann N, Gempt J, Foerschler A, et al (2012) Utility of presurgical navigated transcranial magnetic brain stimulation for the resection of tumors in eloquent motor areas. J Neurosurg 116: 994–1001. 22304452 10.3171/2011.12.JNS111524

[11] Pascual-Leone A, Gates JR, Dhuna A (1991) Induction of speech arrest and counting errors with rapid-rate transcranial magnetic stimulation. Neurology 41: 697–702. 2027485 10.1212/wnl.41.5.697

[12] Wassermann EM, Blaxton TA, Hoffman EA, Berry CD, Oletsky H, et al (1999) Repetitive transcranial magnetic stimulation of the dominant hemisphere can disrupt visual naming in temporal lobe epilepsy patients. Neuropsychologia 37: 537–544. 10340313 10.1016/s0028-3932(98)00102-x

[13] Epstein CM, Lah JJ, Meador K, Weissman JD, Gaitan LE, et al (1996) Optimum stimulus parameters for lateralized suppression of speech with magnetic brain stimulation. Neurology 47: 1590–1593. 8960755 10.1212/wnl.47.6.1590

[14] Lioumis P, Zhdanov A, Makela N, Lehtinen H, Wilenius J, et al (2012) A novel approach for documenting naming errors induced by navigated transcranial magnetic stimulation. J Neurosci Methods 204: 349–354. 22108143 10.1016/j.jneumeth.2011.11.003

[15] Vigneau M, Beaucousin V, Herve PY, Jobard G, Petit L, et al (2011) What is right-hemisphere contribution to phonological, lexico-semantic, and sentence processing? Insights from a meta-analysis. Neuroimage 54: 577–593. 20656040 10.1016/j.neuroimage.2010.07.036

[16] Schuhmann T, Schiller NO, Goebel R, Sack AT (2012) Speaking of which: dissecting the neurocognitive network of language production in picture naming. Cereb Cortex 22: 701–709. 21685399 10.1093/cercor/bhr155

[17] Devlin JT, Watkins KE (2007) Stimulating language: insights from TMS. Brain 130: 610–622. 17138570 10.1093/brain/awl331 PMC1820607

[18] Brennan J, Pylkkanen L (2012) The time-course and spatial distribution of brain activity associated with sentence processing. Neuroimage 60: 1139–1148. 22248581 10.1016/j.neuroimage.2012.01.030

[19] Baumgaertner A, Hartwigsen G, Roman Siebner H (2012) Right-hemispheric processing of non-linguistic word features: Implications for mapping language recovery after stroke. Hum Brain Mapp. 10.1002/hbm.21512 PMC6869885 22359350

[20] Baum SH, Martin RC, Hamilton AC, Beauchamp MS (2012) Multisensory speech perception without the left superior temporal sulcus. Neuroimage. 10.1016/j.neuroimage.2012.05.034 PMC3408546 22634292

[21] Briganti C, Sestieri C, Mattei PA, Esposito R, Galzio RJ, et al.. (2012) Reorganization of Functional Connectivity of the Language Network in Patients with Brain Gliomas. AJNR Am J Neuroradiol. 10.3174/ajnr.A3064 PMC7964610 22555573

[22] Perrone-Bertolotti M, Zoubrinetzky R, Yvert G, Le Bas JF, Baciu M (2012) Functional MRI and neuropsychological evidence for language plasticity before and after surgery in one patient with left temporal lobe epilepsy. Epilepsy Behav 23: 81–86. 22197719 10.1016/j.yebeh.2011.11.011

[23] Bonelli SB, Thompson PJ, Yogarajah M, Vollmar C, Powell RH, et al (2012) Imaging language networks before and after anterior temporal lobe resection: results of a longitudinal fMRI study. Epilepsia 53: 639–650. 22429073 10.1111/j.1528-1167.2012.03433.x PMC4471632

[24] Wang L, Chen D, Yang X, Olson JJ, Gopinath K, et al (2013) Group independent component analysis and functional MRI examination of changes in language areas associated with brain tumors at different locations. PLoS One 8: e59657. 23555736 10.1371/journal.pone.0059657 PMC3608667

[25] Thiel A, Habedank B, Herholz K, Kessler J, Winhuisen L, et al (2006) From the left to the right: How the brain compensates progressive loss of language function. Brain Lang 98: 57–65. 16519926 10.1016/j.bandl.2006.01.007

[26] Thiel A, Habedank B, Winhuisen L, Herholz K, Kessler J, et al (2005) Essential language function of the right hemisphere in brain tumor patients. Ann Neurol 57: 128–131. 15622534 10.1002/ana.20342

[27] Picht T, Krieg SM, Sollmann N, Rosler J, Niraula B, et al (2013) A Comparison of Language Mapping by Preoperative Navigated Transcranial Magnetic Stimulation and Direct Cortical Stimulation During Awake Surgery. Neurosurgery 72: 808–819. 23385773 10.1227/NEU.0b013e3182889e01

[28] Corina DP, Gibson EK, Martin R, Poliakov A, Brinkley J, et al (2005) Dissociation of action and object naming: evidence from cortical stimulation mapping. Hum Brain Mapp 24: 1–10. 15593268 10.1002/hbm.20063 PMC6871733

[29] Sollmann N, Picht T, Makela JP, Meyer B, Ringel F, et al (2013) Navigated transcranial magnetic stimulation for preoperative language mapping in a patient with a left frontoopercular glioblastoma. J Neurosurg 118: 175–179. 23101450 10.3171/2012.9.JNS121053

[30] Rossi S, Hallett M, Rossini PM, Pascual-Leone A (2009) Safety, ethical considerations, and application guidelines for the use of transcranial magnetic stimulation in clinical practice and research. Clin Neurophysiol 120: 2008–2039. 19833552 10.1016/j.clinph.2009.08.016 PMC3260536

[31] Ruohonen J, Ilmoniemi RJ (1999) Modeling of the stimulating field generation in TMS. ElectroencephalogrClinNeurophysiolSuppl 51: 30–40. 10590933

[32] Ilmoniemi RJ, Ruohonen J, Karhu J (1999) Transcranial magnetic stimulation—a new tool for functional imaging of the brain. Crit RevBiomedEng 27: 241–284. 10864281

[33] Ruohonen J, Karhu J (2010) Navigated transcranial magnetic stimulation. NeurophysiolClin 40: 7–17. 10.1016/j.neucli.2010.01.006 20230931

[34] Knops A, Nuerk HC, Sparing R, Foltys H, Willmes K (2006) On the functional role of human parietal cortex in number processing: How gender mediates the impact of a 'virtual lesion' induced by rTMS. Neuropsychologia 44: 2270–2283. 16828812 10.1016/j.neuropsychologia.2006.05.011

[35] Candidi M, Urgesi C, Ionta S, Aglioti SM (2008) Virtual lesion of ventral premotor cortex impairs visual perception of biomechanically possible but not impossible actions. Soc Neurosci 3: 388–400. 18979387 10.1080/17470910701676269

[36] Orosz A, Jann K, Wirth M, Wiest R, Dierks T, et al (2012) Theta burst TMS increases cerebral blood flow in the primary motor cortex during motor performance as assessed by arterial spin labeling (ASL). Neuroimage 61: 599–605. 22613775 10.1016/j.neuroimage.2012.03.084

[37] Salmelin R, Helenius P (2000) Service E (2000) Neurophysiology of fluent and impaired reading: a magnetoencephalographic approach. J Clin Neurophysiol 17: 163–174. 10831107 10.1097/00004691-200003000-00005

[38] Wheat KL, Cornelissen PL, Sack AT, Schuhmann T, Goebel R, et al.. (2012) Charting the functional relevance of Broca's area for visual word recognition and picture naming in Dutch using fMRI-guided TMS. Brain Lang. 10.1016/j.bandl.2012.04.016 22632811

[39] Indefrey P (2011) The spatial and temporal signatures of word production components: a critical update. Front Psychol 2: 255. 22016740 10.3389/fpsyg.2011.00255 PMC3191502

[40] Davidson DJ, Indefrey P (2011) Error-related activity and correlates of grammatical plasticity. Front Psychol 2: 219. 21960979 10.3389/fpsyg.2011.00219 PMC3176590

[41] van de Meerendonk N, Indefrey P, Chwilla DJ, Kolk HH (2011) Monitoring in language perception: electrophysiological and hemodynamic responses to spelling violations. Neuroimage 54: 2350–2363. 20955801 10.1016/j.neuroimage.2010.10.022

[42] Ojemann GA, Whitaker HA (1978) Language localization and variability. Brain Lang 6: 239–260. 728789 10.1016/0093-934x(78)90061-5

[43] Binder JR, Desai RH, Graves WW, Conant LL (2009) Where is the semantic system? A critical review and meta-analysis of 120 functional neuroimaging studies. Cereb Cortex 19: 2767–2796. 19329570 10.1093/cercor/bhp055 PMC2774390

[44] Schwartz MF, Kimberg DY, Walker GM, Faseyitan O, Brecher A, et al (2009) Anterior temporal involvement in semantic word retrieval: voxel-based lesion-symptom mapping evidence from aphasia. Brain 132: 3411–3427. 19942676 10.1093/brain/awp284 PMC2792374

[45] FitzGerald DB, Cosgrove GR, Ronner S, Jiang H, Buchbinder BR, et al (1997) Location of language in the cortex: a comparison between functional MR imaging and electrocortical stimulation. AJNR Am J Neuroradiol 18: 1529–1539. 9296196 PMC8338146

[46] Giussani C, Roux FE, Ojemann J, Sganzerla EP, Pirillo D, et al (2010) Is preoperative functional magnetic resonance imaging reliable for language areas mapping in brain tumor surgery? Review of language functional magnetic resonance imaging and direct cortical stimulation correlation studies. Neurosurgery 66: 113–120. 10.1227/01.NEU.0000360392.15450.C9 19935438

[47] Roux FE, Boulanouar K, Lotterie JA, Mejdoubi M, LeSage JP, et al. (2003) Language functional magnetic resonance imaging in preoperative assessment of language areas: correlation with direct cortical stimulation. Neurosurgery 52: : 1335–1345; discussion 1345–1337. 10.1227/01.neu.0000064803.05077.40 12762879

[48] Shafto MA, Stamatakis EA, Tam PP, Tyler LK (2010) Word retrieval failures in old age: the relationship between structure and function. J Cogn Neurosci 22: 1530–1540. 19642890 10.1162/jocn.2009.21321

[49] Stamatakis EA, Shafto MA, Williams G, Tam P, Tyler LK (2011) White matter changes and word finding failures with increasing age. PLoS One 6: e14496. 21249127 10.1371/journal.pone.0014496 PMC3017545

[50] Geva S, Jones PS, Crinion JT, Price CJ, Baron JC, et al (2012) The effect of aging on the neural correlates of phonological word retrieval. J Cogn Neurosci 24: 2135–2146. 22849403 10.1162/jocn_a_00278 PMC3477855

[51] Smith AE, Ridding MC, Higgins RD, Wittert GA, Pitcher JB (2009) Age-related changes in short-latency motor cortex inhibition. Exp Brain Res 198: 489–500. 19618169 10.1007/s00221-009-1945-8

[52] Tracy JI, Waldron B, Glosser D, Sharan A, Mintzer S, et al (2009) Hemispheric lateralization and language skill coherence in temporal lobe epilepsy. Cortex 45: 1178–1189. 19286172 10.1016/j.cortex.2009.01.007

[53] Cousin E, Baciu M, Pichat C, Kahane P, Le Bas JF (2008) Functional MRI evidence for language plasticity in adult epileptic patients: Preliminary results. Neuropsychiatr Dis Treat 4: 235–246. 18728818 10.2147/ndt.s2330 PMC2515912

[54] Cousin E, Peyrin C, Pichat C, Lamalle L, Le Bas JF, et al (2007) Functional MRI approach for assessing hemispheric predominance of regions activated by a phonological and a semantic task. Eur J Radiol 63: 274–285. 17339089 10.1016/j.ejrad.2007.01.030

[55] Thivard L, Hombrouck J, du Montcel ST, Delmaire C, Cohen L, et al (2005) Productive and perceptive language reorganization in temporal lobe epilepsy. Neuroimage 24: 841–851. 15652319 10.1016/j.neuroimage.2004.10.001

[56] Billingsley RL, McAndrews MP, Crawley AP, Mikulis DJ (2001) Functional MRI of phonological and semantic processing in temporal lobe epilepsy. Brain 124: 1218–1227. 11353737 10.1093/brain/124.6.1218

[57] Demonet JF, Thierry G, Cardebat D (2005) Renewal of the neurophysiology of language: functional neuroimaging. Physiol Rev 85: 49–95. 15618478 10.1152/physrev.00049.2003

[58] Hickok G (2009) The functional neuroanatomy of language. Phys Life Rev 6: 121–143. 20161054 10.1016/j.plrev.2009.06.001 PMC2747108

[59] Goldmann RE, Golby AJ (2005) Atypical language representation in epilepsy: implications for injury-induced reorganization of brain function. Epilepsy Behav 6: 473–487. 15878308 10.1016/j.yebeh.2005.03.012

[60] Galluzzi S, Lanni C, Pantoni L, Filippi M, Frisoni GB (2008) White matter lesions in the elderly: pathophysiological hypothesis on the effect on brain plasticity and reserve. J Neurol Sci 273: 3–9. 18672256 10.1016/j.jns.2008.06.023

[61] Pellicciari MC, Miniussi C, Rossini PM, De Gennaro L (2009) Increased cortical plasticity in the elderly: changes in the somatosensory cortex after paired associative stimulation. Neuroscience 163: 266–276. 19524024 10.1016/j.neuroscience.2009.06.013

[62] Saur D, Lange R, Baumgaertner A, Schraknepper V, Willmes K, et al (2006) Dynamics of language reorganization after stroke. Brain 129: 1371–1384. 16638796 10.1093/brain/awl090

[63] Sarubbo S, Latini F, Sette E, Milani P, Granieri E, et al (2012) Is the resection of gliomas in Wernicke's area reliable? : Wernicke's area resection. Acta Neurochir (Wien) 154: 1653–1662. 22832977 10.1007/s00701-012-1416-z

[64] Robles SG, Gatignol P, Lehericy S, Duffau H (2008) Long-term brain plasticity allowing a multistage surgical approach to World Health Organization Grade II gliomas in eloquent areas. J Neurosurg 109: 615–624. 18826347 10.3171/JNS/2008/109/10/0615

[65] Ius T, Angelini E, Thiebaut de Schotten M, Mandonnet E, Duffau H (2011) Evidence for potentials and limitations of brain plasticity using an atlas of functional resectability of WHO grade II gliomas: Towards a "minimal common brain". Neuroimage 56: 992–1000. 21414413 10.1016/j.neuroimage.2011.03.022

[66] Yordanova YN, Moritz-Gasser S, Duffau H (2011) Awake surgery for WHO Grade II gliomas within "noneloquent" areas in the left dominant hemisphere: toward a "supratotal" resection. Clinical article. J Neurosurg 115: 232–239. 21548750 10.3171/2011.3.JNS101333

